# Delta-proteobacterial SAR324 group in hydrothermal plumes on the South Mid-Atlantic Ridge

**DOI:** 10.1038/srep22842

**Published:** 2016-03-08

**Authors:** Huiluo Cao, Chunming Dong, Salim Bougouffa, Jiangtao Li, Weipeng Zhang, Zongze Shao, Vladimir B. Bajic, Pei-Yuan Qian

**Affiliations:** 1 Division of Life Science, The Hong Kong University of Science and Technology, Clear Water Bay, Hong Kong, China; 2 Key Laboratory of Marine Biogenetic Resources, The Third Institute of Oceanography, State of Oceanic Administration, Xiamen, China; 3 Computational Bioscience Research Center (CBRC), King Abdullah University of Science and Technology (KAUST), Thuwal, Saudi Arabia; 4 State Key Laboratory of Marine Geology, Tongji University, Shanghai, China

## Abstract

In the dark ocean, the SAR324 group of Delta-proteobacteria has been associated with a chemolithotrophic lifestyle. However, their electron transport chain for energy generation and information system has not yet been well characterized. In the present study, four SAR324 draft genomes were extracted from metagenomes sampled from hydrothermal plumes in the South Mid-Atlantic Ridge. We describe novel electron transport chain components in the SAR324 group, particularly the alternative complex III, which is involved in energy generation. Moreover, we propose that the C-type cytochrome, for example the *C*_*553*_, may play a novel role in electron transfer, adding to our knowledge regarding the energy generation process in the SAR324 cluster. The central carbon metabolism in the described SAR324 genomes exhibits several new features other than methanotrophy e.g. aromatic compound degradation. This suggests that methane oxidation may not be the main central carbon metabolism component in SAR324 cluster bacteria. The reductive acetyl-CoA pathway may potentially be essential in carbon fixation due to the absence of components from the Calvin-Benson cycle. Our study provides insight into the role of recombination events in shaping the genome of the SAR324 group based on a larger number of repeat regions observed, which has been overlooked thus far.

The affiliation of the SAR324 cluster bacteria with the Delta-proteobacteria was first proposed based on its 16S rRNA gene classification^1^. Although many studies have included the SAR324 cluster in their evaluations, the results were mostly restricted to surveys of the ecological distribution and phylogenetic diversity^2^,^3^,^4^. The eco-physiology and genetic background of the cluster have not been well characterized. Furthermore, the importance of the metabolic pathways in the SAR324 cluster bacteria has recently been recognized^5^,^6^,^7^,^8^.

The C1 metabolism and a particle-associated life-style of SAR324 cluster bacteria were predicted based on partial genomic sequences^7^. Further studies validated the importance of C1 metabolism in the growth of SAR324. This finding was related specifically to methane, as a high concentration of methane was detected in the hydrothermal plume in the Guaymas Basin^6^,^7^. Microautoradiography and fluorescence *in situ* hybridization further confirmed the uptake of bicarbonate and the particle association of SAR324 bacteria via the detection of ribulose-1,5-bisphosphate carboxylase-oxygenase and sulfur oxidation genes in subtropical gyres^8^. In addition, a diverse array of transporters for organic compounds similar to the SAR324 clade were identified in microbial plankton inhabiting a seasonally hypoxic basin in the Northwest Atlantic Ocean (Bedford Basin), revealing their active heterotrophic lifestyle^5^. However, the mechanisms underlying carbon source utilization in the SAR324 cluster bacteria have not been sufficiently documented, in particular in hydrothermal plumes on middle ocean ridges.

Although the role of SAR324 cluster bacteria in the sulfur and nitrogen cycle has been proposed previously based on the functional genes retrieved from metagenomic or metatranscriptomic data^6^,^7^, the mechanisms underlying the electron generation via the sulfur-utilization process remains unclear. In general, previous studies have suggested that nitrate could be an important electron acceptor for the SAR324 cluster bacteria due to the oxygen depletion in the area^7^,^8^. The components of the entire electron transport chain employed by SAR324 cluster bacteria in the hydrothermal plume of the Guaymas basin have been proposed^7^; however, complete information is still lacking.

In addition, the effects of bacteriophages on planktonic SAR324 cluster bacteria are not well understood. The effects of the viruses on planktonic bacteria could play an important role in the marine cycle, as demonstrated in recent studies that identified functional genes related to the sulfur cycle in the virus that specifically affects the sulfur-oxidizing gamma-proteobacteria SUP05 group^10^,^11^. Therefore, in bacterial groups with unknown or insufficiently known functions, e.g., SAR324 cluster bacteria, the assessment of bacteriophages is important for deciphering the carbon, sulfur or nitrogen cycle in marine environments.

In the present study, genomics bins of SAR324 cluster bacteria were extracted from the metagenomics data generated from three hydrothermal plumes sampled in the South Mid-Atlantic Ridge (SMAR) (Fig. 1 and Table 1). We used several published partial genomes of the SAR324 cluster bacteria generated using a single cell genomics (SCG) method from subtropical gyres^8^ and one genomic bin from the hydrothermal plume in Guaymas basin^6^,^7^ for comparison with those from SMAR.

## Results and Discussion

### Genomic features

In the present study, we extracted genomic bins that belong to the SAR324-cluster bacteria from hydrothermal plume metagenomes from the South Mid-Atlantic ridge (Fig. S1). The GC content of the extracted genomes ranging from 41.3 to 42.4% is similar to those genomes that were reported for others from the Guaymas basin plumes^6^,^7^, subtropical gyres^8^, and some unpublished yet publicly-available genomes (Table 2 and S1). Remarkably, more than 40% of open reading frames (ORFs) in these genomes were hypothetical proteins based on annotation against the SEED subsystem, KEGG, COG (sequence-based) and the Pfam databases (profile-based)^12^ (Table S1).

### Phylogenies of SAR324

The 16S rRNA genes were extracted from contigs that were assigned to two SAR324 genomic bins (only CTD7A-SAR324 and CTD7B-SAR324 had positive results) and constructed into a phylogenetic tree along with other closely-related sequences (Fig. 2). Both SAR324 bacteria formed a clade with uncultured bacteria sharing 99% identity (Fig. 2). Up to now, there is only one partial genome available in GenBank that can be assigned to SAR324 based on its 16S rRNA gene (Delta-proteobacterium SCGC AAA001-C10, HQ675364.1)^8^. The two 16S rRNA gene sequences in the present study also exhibited remote phylogenetic relationship with a 16S rRNA gene with accession U65908.1 (Fig. 2). In contrast, the two 16S rRNA gene sequences that were extracted from metagenomic data from hydrothermal plumes in the Guaymas Basin were grouped with those from previous studies and with some 16S rRNA gene sequences that were retrieved from published partial single-cell genomes^8^, and unpublished data in GenBank (Fig. 2).

### Central carbon metabolism

The SAR324 cluster bacteria are mixotrophs because of their carbon fixation and heterotrophic carbon utilization capabilities^7^. Microautoradiography and fluorescence *in situ* hybridization confirmed the uptake of bicarbonate and the particle association of SAR324 bacteria via the detection of ribulose-1,5-bisphosphate carboxylase-oxygenase and sulfur oxidation genes^8^. However, in the present study, the key genes involved in the Calvin-Benson-Bassham (CBB) Cycle, mainly *cbbL/cbbM*, were absent in all of the SAR324 group genomic bins from SMAR but were present in SCGC-AAA001-C10, AB-629-O05, and GB-SAR324 (Table S2). However, phylogenetic analysis of the 16S rRNA gene revealed close relationship between the SAR324 bacteria from this study and SCGC-AAA001-C10 (Fig. 2). The phylogenetic analysis based on CbbL/CbbM protein sequences supported different affiliations for the *cbbL* genes from genomic bins in the present study (Fig. S1). Those from SCGC-AAA001-C10 and AB-629-O05 were assigned to form I *cbbL* (close to the Gamma-proteobacteria), while GB-SAR324 was affiliated with form II *cbbM* (close to the Alpha-proteobacteria) (Fig. S1). It is possible that these *cbbL/M* genes originated via horizontal gene transfer.

The genes that encode carbon monoxide dehydrogenase and acetyl-CoA synthase, which catalyze the reductive acetyl coenzyme A pathway (Wood–Ljungdahl pathway), could play a main role in carbon fixation metabolism rather than the CBB cycle identified in other SAR324 (Fig. 3 and Table S2). This pathway has generally been overlooked in previous studies of the SAR324 cluster bacteria^7^,^8^. A phylogenetic analysis of the gene encoding acetyl-CoA synthase suggests that two types of enzymes are present in some strains; however, only one type was detected in SMAR SAR324 bacteria (Fig. S2). The close relationship between the acetyl-CoA synthase genes hints an intriguing evolutionary history within SAR324 cluster bacteria (Fig. S2).

Heterotrophic pathways seem to be more important than autotrophic pathways in the SAR324 cluster bacterial growth (Fig. 3 and Table S2). This is the first study to demonstrate the presence of the aromatic compound metabolism pathway in the SAR324 bacteria, and its exclusive presence in the SMAR strains^7^,^8^,^9^. Several genes that encode enzymes capable of degrading aromatic compounds were identified in the extracted SAR324 genomic bins. In the toluene and naphthalene degradation pathways, oxygenase was missing, and only two components were identified in the naphthalene and biphenyl pathways (Table S2). Another conserved gene cluster was identified in all of the SAR324 cluster bacteria and was annotated as nitrilotriacetate monooxygenase component B (EC 1.14.13.-) but function of this cluster is unclear. Isoquinoline 1-oxidoreductase was found in the current SAR324 draft genomes as well as in the partial SCGs (SCGC AAA001-C10 and JCVI-SC AAA005) included here. Isoquinoline 1-oxidoreductase that catalyzes N-heterocyclic aromatic compound degradation and absent in other SAR324 cluster bacteria (Table S2). To rule out the possibility of a chimeric contig, the taxonomic assignment of the flanking regions of the isoquinoline 1-oxidoreductase genes were also checked.

Many of the benzoate degradation pathway genes were detected including the inner-membrane benzoate translocator (Fig. 3 and Table S2). A nearly complete set of genes for the benzoyl-CoA degradation pathway was detected but our findings differed from previous reports^13^,^14^. In all of the SMAR-SAR324 genomic bins, one conserved gene cluster that includes benzoyl-CoA ligase, benzoyl-CoA oxygenase component B (BoxB), and benzoyl-CoA dihydrodiol lyase (BoxC), all of which are a part of the aerobic benzoyl-CoA degradation pathway (13,14). All of these genes were only identified in SAR324 genomic bins from the SMAR and not in any other SAR324 bacteria surveyed to date (Fig. 3 and Table S2). However, 3,4-dehydroadipyl-CoA semialdehyde dehydrogenase/NADP^+^-specific aldehyde dehydrogenase (BoxD), which is responsible for the conversion of 3,4-didehydroadipyl-CoA semialdehyde to 3,4-didehydroadipyl-CoA in *Azoarcus evansii*^15^ and *Burkholderia xenovorans*^16^, was not detected in any of the genomic bins in the present study and referenced data (Fig. 3 and Table S2). Considering that several NADP^+^-dependent aldehyde dehydrogenases were detected (Table S2), an alternative pathway might be responsible for converting 3,4-didehydroadipyl-CoA semialdehyde to 3,4-didehydroadipyl-CoA or other intermediates. Via this process, formate was also produced in the step catalyzing the conversion of 2,3-epoxybenzoyl-CoA to 3,4-didehydroadipyl-CoA semialdehyde by benzoyl-CoA dihydrodiol lyase (BoxC).

The intermediate products derived from the process described above could then be transferred into the beta-oxidation pathway. The gene that encodes the beta-ketoadipyl-CoA thiolase (BoxE) responsible for converting beta-ketoadipyl CoA to acetyl-CoA and succinyl-CoA was absent in all of the genomic bins (Fig. 3). However, one ketoacyl-CoA thiolase was identified, and it was distinct from the other ketoacyl-CoA thiolases (Fig. S3). Interestingly, although an aerobic aromatic degradation pathway was identified as previously mentioned, some relatives of the deduced putative proteins associated with the partial anaerobic pathway, including 3-oxoadipyl-CoA/3-oxo-5,6-dehydrosuberyl-CoA thiolase (PaaJ), were also present in the SAR324 cluster bacterial genomic bins (Fig. 3 and Table S2). In addition, 3-hydroxyadipyl-CoA dehydrogenase (PaaH) was found (Fig. S4). The PaaL gene was also detected in the CTD7A-SAR324 genomic bin. PaaL gene shares 99% identity with a hypothetical protein of the Deltaproteobacterium SCGC AAA003-F15 (WP_029734952.1) but bears little similarity to other reported genes (<57% identity on protein level). This finding supports the ubiquity of PaaL in SAR324 cluster bacteria.

4-hydroxybenzoyl-CoA reductase and thioesterase were identified in the CTD10-SAR324 and CTD10-SAR324 genomic bins (Table S2). These components could form the side chain pathway for the degradation of aromatic compounds to produce 4-hydroxybenzoate and interact with the tyrosine metabolism pathway. Moreover, one alpha/beta hydrolase family protein that is closely involved in the benzoate degradation ring-cleavage hydrolase or 2-succinyl-6-hydroxy-2,4-cyclohexadiene-1-carboxylate synthase was identified in all of the genomic bins from the present study, but not in CTD7A-SAR324-2 (Fig. 3 and Table S2). The function of this enzyme has not been determined yet (Fig. S5).

In previous studies investigating SAR324 cluster bacteria from the Guaymas Basin, particulate hydrocarbon monooxygenases (pHMO, such as particulate methane monooxygenase) were identified^6^. The pHMO genes were not identified in all of the long contigs, but only in the short contigs in that study^7^. One gene cluster encoding pHMO was identified in one genome bin (SCGC_AAA240_J09). However, the pHMO genes were not identified in all of the contigs assigned to SAR324 in the present study and other data. This result indicates that pHMO could potentially play a minor role, or that other enzymes may play a more important role, in C_1_ or C_2_-C_4_ hydrocarbon utilization.

Overall, carbon fixation may play a minor role in the carbon supply for the growth of SAR324 cluster bacteria, while versatile carbon compounds could function as electron donors through a batch of dehydrogenases to feed SAR324 cluster bacteria as are discussed below.

### Energy conservation via the electron transport chain

The main components involved in the electron transport chain, which include NADH-ubiquinone oxidoreductase and cytochrome *c* oxidase, were identified. However, the canonical cytochrome oxidoreductase was not found (Fig. 3 and Table S3). Instead, several uncharacterized oxidoreductases were identified. Overall, the periplasmic enzymes were less abundant in SAR324 cluster bacteria, suggesting a minor role for this type of energy conservation process in comparison to the cytoplasmic route of electron transfer.

Several multi-heme *c*-type cytochromes (MHCs), which are metalloproteins with various functions in the catalysis of substrates and in electron transfer, were present in the SAR324 genomic bins in the present study. Among these MHCs, soluble cytochrome *C*_*553*_ was identified in all of the SAR324 genomic bins and was predicted to be the electron transfer partner of formate dehydrogenase (FDH) and of [Fe]-hydrogenase, which are essential components in the metabolism of sulfate-reducing bacteria^17^ (Fig. 3 and Table S3). The percentage identity at the amino acid level was 99% with the cytochrome *C*_*553*_ from SCGCAAA001-C10 SAR324 cluster bacteria; but it remains distant from the other sequences deposited in GenBank with an identity of less than 59%. These results support the ubiquity of this electron transfer process via cytochrome *C*_*553*_ in SAR324 cluster bacteria (Fig. S6). However, *C*_*553*_ was not recovered from the SAR324 cluster genomic bin from the Guaymas Basin hydrothermal plume. Instead, five copies of cytochrome *C*_*4*_ were present in tandem, suggesting an alternative route of electron transfer. Interestingly, the genes flanking *C*_*553*_ had unknown functions. Considering the fewer number of hydrogenases and the presence of formate dehydrogenase, the electron transport route was predicted to proceed from formate to cytochrome *C*_*553*_, which is consistent with the formate produced during aromatic degradation (Table S2). In a previous study^7^, the periplasmic-oriented, membrane-associated formate dehydrogenase was proposed to be linked to the quinone pool through a membrane-spanning polysulfide reductase (NrfD)^18^. NrfD was present in the SAR324 bacteria genomic bins in the present study as several types of NrfD were identified (Fig. 3, Table S3). All of the NrfDs detected in the present study and in other SAR324 genomic bins clustered together and were closely related to the alternative complex III (cytochrome oxidoreductase) protein (ActC) annotated in *Ignavibacterium album* (51% identity)^19^, *Gemmatimonadetes* bacterium KBS708^20^, and Candidate Division ZIXI available in GenBank (unpublished), while they were also closely related to the hydrogenase in *Gemmatimonas* sp. AP64 (49% identity)^21^ (Fig. S7). Considering the absence of complex III (cytochrome *c* oxidoreductase) in all of the genomic bins, this type of NrfD might be responsible for the cytochrome reduction process as observed in *Ignavibacterium album*^19^.

Of the two polysulfide reductases identified in the present study (Fig. S7), one exhibited low identity to those reported to date (<32% on protein level), particularly in relation to the archaeal polysulfide reductase e.g., *Ferroglobus placidus*, with the exception of one hypothetical protein ETSY2_41500 in Candidatus *Entotheonella* sp. TSY2 (53% identity on protein level)^22^. The other polysulfide was reported previously^7^ and is responsible for the transfer of electrons to the quinone pool from formate dehydrogenase (Fig. S7). The electron transfer flavoprotein (ETF was constituted of two subunits and is responsible for the transfer of electrons from hydrogenase to terminal respiratory systems. ETF was found in CTD7A-SAR324, CTD10-SAR324, GB-SAR324 and SCGC_AB_629_J17, but the absence of hydrogenase in all SAR324 cluster bacteria suggests an alternative role for this ETF.

Cytochrome *C*_*4*_ was identified in all of the genomic bins (Fig. 3 and Table S3), but the comparison analysis revealed a remote phylogenetic relationship of the SAR324 cluster bacteria from the present study and the referenced partial genomes with other groups deposited in GenBank (<61%) (Fig. S8). Cytochrome *C*_*4*_ is an intermediate in the transfer of electrons to the terminal oxidase in *Vibrio choleraae*^23^. Five copies of cytochrome *C*_*4*_ in tandem were identified in the genomic bin from the hydrothermal plume in the Guaymas basin, and two copies were identified in the SCGs SCGC_AB_629_O05 and SCGC_AAA001_C10. However, in two other SCGs, SCGC_AAA005 and SCGC_AAA240_J09, no cytochrome *C4* was identified. Instead, other cytochrome c proteins were identified, including additional copies of cytochrome *C*_*553*_ in both SCGs and one cytochrome *C*_*551*_ in SCGC_AAA240_J09 (Fig. 3 and Table S3). Cytochrome oxidase was also recovered from all of the genomic bins along with the assembly proteins (Table S3).

Reduced organic sulfur compounds can serve as electron donors for SAR324 cluster bacteria with energy conversion processes via the dissimilatory sulfate reduction pathway^7^,^8^. However, this pathway is incomplete in SAR324 from SMAR and in other genomic bins (Fig. 3 and Table S3). Only genes encoding sulfate adenylyltransferase (*sat*), heterodisulfide reductase (*hdr*) and adenylyltransferase reductase (*apr*) were identified in CTD7B-SAR324 within the same cluster but were completely absent in the other SAR324 genomic bins (CTD7A-SAR324 and CTD10-SAR324) (Table S3). In addition, a similar gene cluster was identified in SCGC_AAA240_J09 SCG and displayed an identical gene arrangement. Interestingly, this gene cluster from SCGC_AAA240_J09 SCG is located on the same contig as SoxB and SoxZ, suggesting the potential co-translation of these genes to execute the sulfur oxidation process. Remarkably, sulfide dehydrogenase (flavocytochrome C) was only identified in SAR324 bacteria from SMAR; CTD7A-SAR324 and CTD10-SAR324 but not in CTD7B-SAR324 or the referenced data (Table S3). Moreover, the phylogenetic analysis revealed the phylogenetic position of this gene, which clustered with one from the unpublished single genome of the Delta-proteobacteria bacterium SCGC AAA003-J15 deposited in GenBank with 97% identity. However, its identity with all of the other proteins deposited in GenBank was lower than 57% (Fig. S9). The function of the sulfide dehydrogenase (flavocytochrome C) in SAR324 cluster bacteria may be related to sulfur oxidation because the flanking regions of the gene were annotated as sulfur oxidation-related proteins (SoxY and SoxZ) albeit with a low identity (<53% at the amino acid level) (Fig. S10). However, further analysis did not result in the recovery of additional sox complex subunits in the CTD7A-SAR324 or the CTD10-SAR324 genomic bins. In conclusion, the function of flavocytochrome C identified in CTD7A-SAR324 and CTD10-SAR324 could be related to the reduced sulfur compound oxidation and thus may represent a novel mechanism coupled with an incomplete sox complex (soxYZ subunits) that differs from the conventional sulfur oxidation pathway via the sox complex.

One orphan copper-containing nitrite reductase (EC 1.7.2.1) involved in the denitrifying process was identified in SCGCAAA001-C10 SAR324 cluster bacteria. In addition, within the single cell genome of the SCGCAAA001-C10 SAR324 cluster bacteria, one NnrU family protein, which is required for the expression of nitric oxide and nitrite reductases (Nir and Nor) was identified in one contig. This contig contained a phage island that was proposed to feature phage proteins, e.g., integrase or the MORN repeat region suggesting the foreign origin of NnrU. However, no genes were assigned to the denitrifying pathway in any of the genomic bins from SMAR, which differs from all of the other SAR324 cluster bacteria reported previously^7^,^8^. Overall, these facts indicate the minor role of nitrogen compounds as electron acceptors.

### Chemotaxis and stress response

The chemotactic lifestyle of SAR324 cluster bacteria has not been discussed in detail to date^7^,^8^, but it is important for understanding the ecological function of this newly discovered but not well documented bacterial cluster. Within the chemotaxis-associated characteristics in genomic structures, we observed an over-representation of genes encoding flagellar-related assembly proteins (Table S4), but these genes were scattered throughout the entire genome rather than being localized in regions adjacent to the co-transcript. In addition, a greater number of hypothetical proteins were identified adjacent to flagellar-encoding genes in the SAR324 cluster bacterial genomic bins. For instance, in one contig from the SAR324-CTD7B genomic bin possessing the flagellar synthesis regulator FleN protein, the neighboring genes were initially annotated as hypothetical proteins; however, further studies showed that most of the genes were conserved exclusively in SAR324 cluster bacteria (with 95-98% identity at the amino acid level) indicating conserved function of these hypothetical proteins in SAR324.

Regarding the planktonic lifestyle of SAR324 cluster bacteria in marine water column, the fine-scale regulation or communication with other types of bacteria should necessitate the acquisition of more favorable substrates to support growth. However, the genes that encode chemotaxis proteins were not abundant or were even absent in comparison to GB-SAR324 which has a relatively complete set of chemotaxis genes (Table S4). Only genes associated with flagellar motor proteins were recovered in SMAR-SAR324 in numbers that were consistent with the high abundance of genes identified in that group of bacteria, suggesting a strong gliding capability (Table S4). Interestingly, genes that encode chemotaxis components were over-represented in the genomic bin JCVI-SC AAA005.

SAR324’s environmental response to the harsh surroundings is likely versatile. One conserved gene cluster responsible for aerotolerance, an operon that includes BatA, TPR and MoxR-like ATPase, PA3071 and other hypothetical proteins, was found in all of the SMAR genomic bins and partially in GB-SAR324 (Fig. S11)^24^, suggesting the involvement in oxygen tolerance, which is consistent with the presence of electron acceptors in the SAR324 genomic bins in the present study.

Repeat regions were observed in abundance in the CTD10-SAR324 genomic bins, which may have resulted in assembly fragmentation (Table 2). The abundance of repeat regions (e.g., 3.48% of all of the predicted genes in CTD10-SAR324) was higher than those in closely related genomic bins, even in CTD7A-SAR324 and CTD7B-SAR324, as well as in other genomic bins from the Guaymas basin plumes^6^,^7^ and in subtropical gyres^8^. As reported before, recombination events challenge the stability of the bacterial genome^25^. Major rearrangements in bacterial genomes are thought to frequently occur via homologous recombination between inverted repeats, and the low repeat number is then frequently associated with genome stability^26^. However, additional repeat regions could also be one mechanism of adaptation to harsh environments to acquire beneficial mutations. The large amount of repetitive sequences observed in CTD10-SAR324 cluster bacteria suggests that the genome is currently very dynamic.

In summary, the present study proposed novel electron transfer optional routes for energy conservation in SAR324 cluster bacteria. Specifically, we discovered one alternative complex III protein, cytochrome *C*_*553*_ and *C*_*4*_. Moreover, we extend the present understanding of carbon utilization in SAR324 cluster bacteria, especially in terms of aromatic compound degradation and the reductive acetyl-CoA pathway. Furthermore, methanotrophy might not be well supported in SAR324 cluster bacteria like it is in some SAR324 bacteria from Guaymas basin hydrothermal plumes. Our present study also provides insight regarding the role of repeat regions in shaping genomes of SAR324 cluster bacteria.

## Methods

### Samples collected in the South Mid-Atlantic and molecular experiments

**Figure 1:**
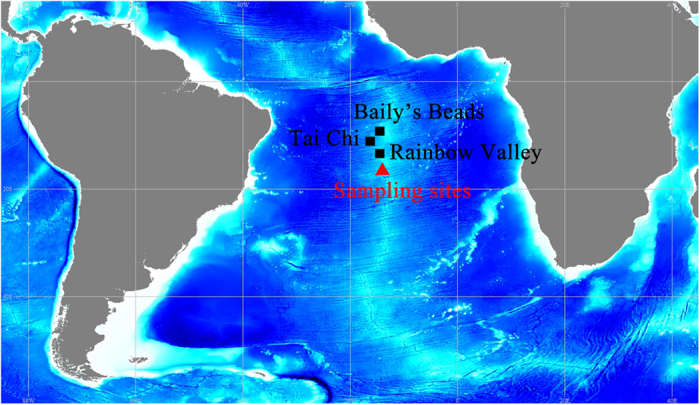
Sampling sites of hydrothermal plumes on the South Mid-Atlantic Ridge with three referenced hydrothermal fields reported previously. Bathymetric imagery was derived from the GEBCO_2014 Grid, www.gebco.net.

**Figure 2:**
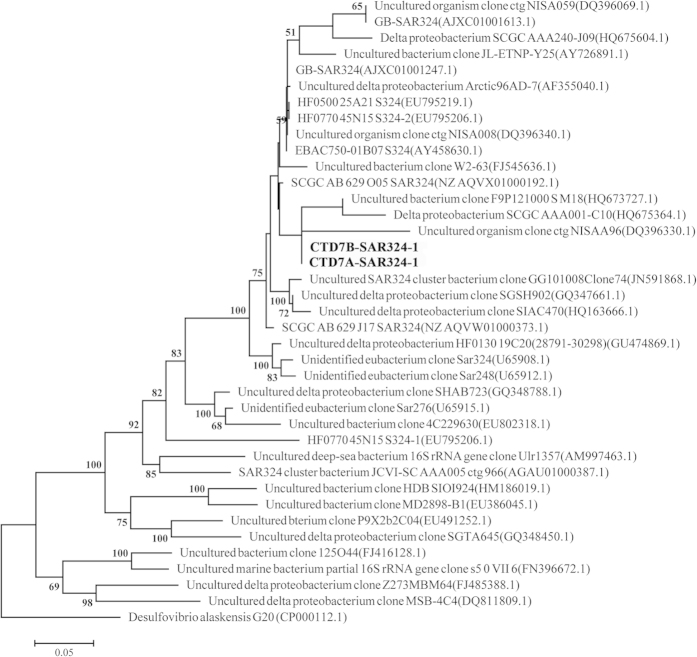
Phylogenetic tree deduced from 16S rRNA gene sequences in bold retrieved from SAR324 cluster bacteria genomic bins in the present study and referenced sequences. The results were obtained using MEGA with ML criteria, and statistical support for each node was calculated using 100 bootstrap replicates.

**Figure 3:**
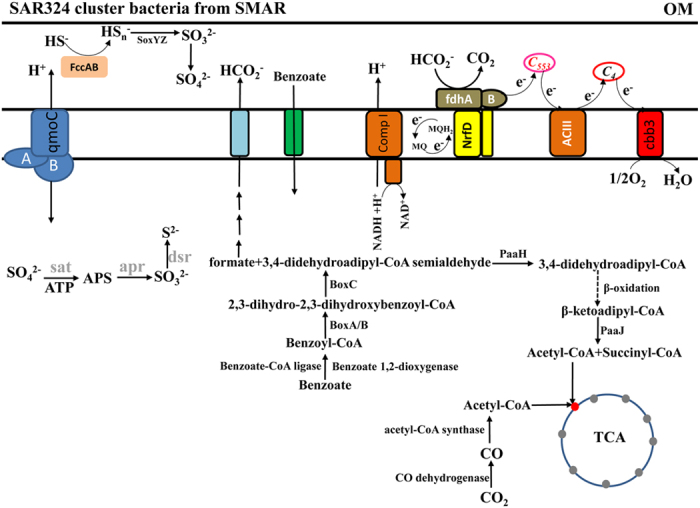
Main schematic metabolic pathways and novel components involved in the electron transport chain in SAR324 cluster bacteria deduced in the present study. NfrD, membrane-spanning polysulfide reductase; ACIII, alterative complex III (cytochrome oxidoreductase) protein; fcc, flavocytochrome C; sox, sulfur oxidation complex; fdh, formate dehydrogenase; sat, sulfate adenylyltransferase; apr, adenosine 5′-phosphosulfate reductase; dsr, dissimilatory sulfite reductase; BoxB, benzoyl-CoA oxygenase component B; BoxC, benzoyl-CoA dihydrodiol lyase; PaaJ, 3-oxoadipyl-CoA/3-oxo-5,6-dehydrosuberyl-CoA thiolase ; PaaH, 3-hydroxyadipyl-CoA dehydrogenase.

In general, all of the samples were collected by a Conductivity, Temperature and Depth (CTD) rosette aboard the “Dayang Yihao” during a DY26 cruise in August 2012 organized by COMRA (China Ocean Mineral Resources R & D Association) on the SMAR (Fig. 1, Table 1). Hydrothermal activity was proposed based on methane and temperature anomalies using additional facilities such as the Portable MAPR (Miniature Autonomous Plume Recorders attached to a towed deep-sea instrument). In addition, hydrothermal chimneys were also observed below the hydrothermal plume sampling sites using cameras bound to towed deep-sea instruments or TV grabbers. Three plume samples (the volume of each sample is shown in Table 1) were collected and filtered with 0.2-μm membranes on board, followed by immediate freezing in liquid nitrogen and preservation at −80 °C until use.

### Environmental DNA extraction and sequ`encing

For each sample, three equal-sized pieces of membrane were subjected to crude genomic DNA extraction according to the following procedure. After homogenization using a sterilized mortar and pestle to release microorganisms and three freeze-thaw cycles, the samples were collected and transferred into new tubes. The filtrates were then collected by centrifugation at 10,000 × *g* for 5 min and stored in 4 ml of DNA extraction buffer. DNA separation was performed as follows. In brief, 50 μl of lysozyme (100 mg/ml) was added to lyse the cells, and then 400 μl of 20% SDS and 40 μl of proteinase K (10 μg/μl) were added for digestion. Chloroform-isoamyl alcohol at a ratio of 24:1 was used to separate the DNA from the mixture, and the DNA was then precipitated with an equal volume of 100% isopropanol. After being washed with cold 75% ethanol, the quantity and quality of the DNA were determined using a Nanodrop device ND2000 (Thermo Scientific, Wilmington, DE, USA) and gel electrophoresis. For each sample, approximately 200 ng of DNA was subjected to an Illumina Hiseq2000 platform (PE500 library) according to the manufacturer’s instructions.

### Bioinformatics procedure

Prior to further genome extractions and annotations, the NGS QC Toolkit (version 2.3) was employed to conduct quality control for the raw Illumina paired-end reads (2 × 150 bp)^27^ (33) by removing low quality sequences (average quality score <20). The first 10 bases of each read and homopolymers (>6 bases) were also trimmed. High-quality reads of all samples were separately assembled using SPAdes version 3.0.1^28^ (34) with parameters that mainly included kmer values ranging from 21 to 85.

After filtering contigs shorter than 500 bp, the remaining contigs were used for gene prediction using Prodigal with the “meta” setting option^29^. Predicted protein sequences were subjected to BLASTp analysis against the updated NCBI non-redundant (nr) database with an *E*-value cutoff of 1e-5 and a maximum hit number of 20. The output from the NR comparison was imported into MEGAN 5 to generate taxonomic information for each contig with the lowest common ancestor (LCA) parameter of minimum support, minimum score maximum expect (0.01), and 5 top percent applied^30^. In addition, qualified short reads from each metagenome were aligned with the long assembled contigs (>500 bp) using Bowtie2^31^. The sequencing coverage of the contigs in each sample was calculated using SAMtools^32^. Information regarding the contig coverage, tetranucleotides and GC content for each contig was calculated via Perl scripts^33^. All of the information for the contigs from the above analyses was integrated and subjected to one pipeline for target genome extraction as described previously^33^,^34^. After obtaining the genomes of SAR324 cluster bacteria, several other partial genomes were also retrieved from GenBank (Table S1) for further comparison studies.

Contigs assigned to SAR324 cluster bacteria genomic bins were extracted and confirmed using an established procedure. Briefly, the genes were mapped to contigs by Prodigal^29^ using the ‘meta’ method, and then BLASTp was applied against the NCBI non-redundant protein database for each genomic bin of SAR324 cluster bacteria. The taxonomic affiliations of all of the BLAST hits were determined using MEGAN 5^30^, and only those contigs with at least half of the predicted proteins assigned to SAR324 cluster bacteria were subjected to the following annotation analyses. Relative abundance of the binned SAR324 was estimated by read recruitment using bowtie2^31^ and fr-hit^35^. The reads were recruited to the three assembled metagomes as well as to the four binned SAR324 genomes. The abundance was derived from the percentage of successfully recruited reads to the binned genomes divided by the percentage of reads that recruited to the assembled metagenomes. All steps were carried out with default settings for bowtie2’s two stages (index building and alignment) and fr-hit. Fr-hit is able to successfully recruit more reads to the assembled contigs compared to bowtie2 and henceforth, we only refer to results obtained with fr-hit.

For all the contigs assigned in this way to SAR324 cluster bacteria, Prokka packages were used to perform the annotations^36^. In addition, several databases including KEGG (http://www.genome.jp/kegg)^37^ and the Clusters of Orthologous Group (COG) sequence database^38^ within the STRING database (v 9.0) (http://string-db.org) were employed to annotate the genes detected in the contigs via Prodigal. Moreover, an HMM search was performed against the Pfam database^12^ using hmmsearch 3.0 with the trusted cutoff for each protein family. To confirm the annotations, a functional classification system SEED hierarchy analysis was conducted online, as well as to determine the function based on the predicted proteins in the contigs (http://rast.nmpdr.org/)^39^. To compare all of the referenced data listed in Supplementary Table S1, all of the pipelines used for the genomic bins from the present study were also applied to these referenced data.

Although no complete genome has been used as a reference to date, for large contig alignments between different genomic bins, Mauve v2.3.1 with default settings^40^ was used to suggest distinct regions at the genome level between these genomic bins and the referenced genomes or contigs.

The 16S rRNA gene fragments from all the genomic bins were predicted using Meta-RNA rRNA prediction^41^ and combined with the referenced sequences from GenBank and the genomes to construct a phylogenetic tree using MEGA v6.05 with maximum-likelihood (ML) criteria and node support with 500 bootstrap replicates^42^. Moreover, a set of phylogenetic analyses was conducted to evaluate the key genes in metabolic pathways, including *cbbL/M*, acetyl-CoA synthase, 3-ketoacyl-CoA thiolase, 3-hydroxyadipyl-CoA dehydrogenase (PaaH), benzoate degradation ring-cleavage hydrolase, cytochrome *C*_*553*_, polysulfide reductase (NfrD), cytochrome *C*_*4*_, and sulfide dehydrogenase (flavocytochrome C). For each gene, the amino acid sequences were aligned to other protein sequences collected from NCBI using MUSCLE3.5^43^. The alignments were checked manually and then used to reconstruct neighbor-joining (NJ) trees with 1000 bootstrap replicates using MEGA. An ML phylogenetic tree was reconstructed based on 500 bootstrap replicates using the consensus NJ tree in MEGA.

### Nucleotide sequence accession number

The raw metagenomic data to extract partial genomes of SAR324 cluster bacteria from the SMAR was submitted to GenBank under accession number PRJNA276313.

## Additional Information

**How to cite this article**: Cao, H. *et al.* Delta-proteobacterial SAR324 group in hydrothermal plumes on the South Mid-Atlantic Ridge. *Sci. Rep.* **6**, 22842; doi: 10.1038/srep22842 (2016).

## Supplementary Material

Supplementary Information

## Figures and Tables

**Table 1:** Samples employed in the present study with metagenomic data information.

Sample ID	Sampling date	Location	Depth(m)	Methane anomaly	Filtered volume (L)	Data size (Gbp)	Assembled contig size (Mbp)	N50	Maximum sequence length (bp)	Average sequence length (bp)
CTD07A	08/02/2012	13.35°W,15.16°S	2600	No	26.2	4	189.69	494	123676	472.64
CTD07B	08/02/2012	13.35°W,15.16°S	2750	Yes	26.2	4	625.63	480	242995	457.80
CTD10B	08/04/2012	13.35°W,15.16°S	2500	Yes	35.6	4	470.39	379	210715	410.13

**Table 2:** Features of the nearly complete genomes of SAR324 cluster bacteria binned from three metagenomes in the present study.

Genome symbol	Total length (Mb)	Number of Contigs	Mean length (kb)	Longest contig (kb)	Relative abundance (bowtie2/fr-hit) (%)	GC (%)	Total essential gene number	Unique essential gene number
CTD07A-SAR324-1	2.21	240	9.21	61.12	1.78/1.85	42.3	98	96
CTD07A-SAR324-2	1.42	158	9.00	46.23	1.14/1.85	41.3	74	72
CTD07B-SAR324-1	2.80	148	18.95	73.92	5.06/5.69	42.3	102	101
CTD10-SAR324	2.93	188	15.60	56.57	2.04/2.59	42.4	102	100
