# Supplementary material for: Delta-proteobacterial SAR324 group in hydrothermal plumes on the South Mid-Atlantic Ridge
Source: Sci Rep. 2016 Mar 8;6:22842. doi: 10.1038/srep22842 (PMC4782131; doi:10.1038/srep22842)
Supplement: Supplementary Information [file srep22842-s1.doc]

**Delta-proteobacterial SAR324 group in hydrothermal plumes on the South Mid-Atlantic Ridge**

Huiluo Cao^1^, Chunming Dong^2^, Salim Bougouffa^3^, Jiangtao Li^4^, Weipeng Zhang^1^, Zongze Shao^2^, Vladimir B. Bajic^3^, Pei-Yuan Qian^1*^

^1^Division of Life Science, The Hong Kong University of Science and Technology, Clear Water Bay, Hong Kong, China, ^2^Key Laboratory of Marine Biogenetic Resources, The Third Institute of Oceanography, State of Oceanic Administration, Xiamen, China,^3^Computational Bioscience Research Center (CBRC), King Abdullah University of Science and Technology (KAUST), Thuwal, Saudi Arabia, ^4^State Key Laboratory of Marine Geology, Tongji University, Shanghai, China

**Running title**: SAR324 group from plumes on the South Mid-Atlantic Ocean Ridge

**Keywords**: SAR324, hydrothermal plume, South Mid-Atlantic Ridge, metagenomic, Illumina

*Corresponding author:

Pei-Yuan Qian, PhD

Division of Life Science

The Hong Kong University of Science and Technology

Clear Water Bay, Hong Kong, China

Phone: 0852-2358-7331

Fax: 0852-2358-1559

E-mail: [boqianpy@ust.hk](mailto:boqianpy@ust.hk)

**Supplementary materials**

Table S1. General information of the incomplete genomic sequences of SAR324 cluster bacteria described in the present study.

| **Label** | **RefSeq accession #** | **Size (Mb)** | **GC (%)** | **Protein** | **rRNA** | **tRNA** | **Other RNA** | **Gene** | **Pseudogene** | **Hypothetical protein(%)** | **Completeness** | **Ref.** |
| --- | --- | --- | --- | --- | --- | --- | --- | --- | --- | --- | --- | --- |
| SCGC AAA240-J09 ASM21335v2 | NZ_AFIA00000000.1 | 2.26 | 41.5 | 1,877 | 3 | 25 | 0 | 1,959 | 54 | 37.7 | 41.35% | Swan et al., 2011 |
| SCGC AB-629-O05 ASM37580v1 | NZ_AQVX00000000.1 | 0.86 | 40.7 | 741 | 3 | 13 | 0 | 768 | 11 | 39.3 | 23.75% | Swan et al., 2011 |
| SCGC AB-629-J17 ASM37578v1 | NZ_AQVW00000000.1 | 1.39 | 39.3 | 1,244 | 3 | 33 | 0 | 1,315 | 35 | 52.0 | 19.05% | Swan et al., 2011 |
| JCVI-SC AAA005 sar324_mda | NZ_AGAU00000000.1 | 4.26 | 43.3 | 3,363 | 3 | 32 | 1 | 3,518 | 119 | 47.3 | 64.21% | Swan et al., 2011 |
| SCGC AAA001-C10 ASM21333v2 | NZ_AFIB00000000.1 | 2.16 | 41.1 | 2,060 | 2 | 21 | 0 | 2,118 | 35 | 36.7 | 49.06% | Swan et al., 2011 |
| GB_SAR324 | AJXC00000000.1 | 2.75 | 41.3 | 3,354 | 3 | 34 | 0 | 3,388 | 245 | 36.0 | 78.68% | Shiek et al., 2013 |
| SCGC AAA003-F15 | NZ_AXVT00000000.1 | 1.06 | 41.3 | 944 | 3 | 20 | 1 | 996 | 28 | 38.7 | 36.46% | Unpublished |
| CTD07A-SAR324-1 | SAMN04448862 | 2.21 | 42.3 | 1,927 | 0 | 16 | 0 | 1,944 | 13 | 40.0 | 91.6% | This study |
| CTD07A-SAR324-2 | SAMN04448863l | 1.42 | 41.3 | 1,257 | 0 | 13 | 0 | 1,270 | 10 | 44.7 | 69.2% | This study |
| CTD07B-SAR324 | SAMN04448864 | 2.80 | 42.3 | 2,446 | 3 | 34 | 0 | 2,481 | 20 | 41.2 | 95.3% | This study |
| CTD10-SAR324 | SAMN04448865 | 2.93 | 42.4 | 2,563 | 1 | 29 | 0 | 2,592 | 20 | 40.1 | 95.3% | This study |

Table S2. Genes involved in carbon metabolism in the SAR324 cluster bacteria described in the present study.

|  | **CTD7A-SAR324-1** | **CTD7A-SAR324-2** | **CTD7B-SAR324** | **CTD10-SAR324** | **GB-SAR324** | **SCGC AAA001-C10** | **JCVI-SC AAA005** | **SCGC AAA240-J09** | **SCGC AB-629-J17** | **SCGC AB-629-O05** |
| --- | --- | --- | --- | --- | --- | --- | --- | --- | --- | --- |
| ***cbbL/M*** | **-** | **-** | **-** | **-** | **+ (form II)** | **+ (form I)** | **-** | **+ (form I)** | **-** | **-** |
| **Acetyl-CoA synthase** | **+** | **-** | **+** | **+** | **+** | **-** | **+** | **+** | **-** | **-** |
| **CO dehydrogenase** | **+** | **+** | **+** | **+** | **-** | **-** | **-** | **-** | **-** | **-** |
| **Aromatic degradation pathway** |  |  |  |  |  |  |  |  |  |  |
| **Succinyl-CoA:(R)-benzylsuccinate CoA-transferase subunit (BbsF)** | **+** | **+** | **+(2)** | **+** | **+** | **+** | **-** | **+** | **-** | **-** |
| **(R)-benzylsuccinyl-CoA dehydrogenase (BbsG)** | **-** | **-** | **-** | **-** | **-** | **-** | **-** | **+** | **+** | **-** |
| **Naphthalene 1,2-dioxygenase system ferredoxin subunit (NdoA)** | **-** | **-** | **-** | **-** | **-** | **-** | **+** | **-** | **-** | **-** |
| **2-hydroxychromene-2-carboxylate isomerase (NahD)** | **+** | **-** | **+(3)** | **+(4)** | **-** | **+** | **+** | **+(2)** | **-** | **+(2)** |
| **Biphenyl dioxygenase subunit alpha (BphA)** | **+** | **-** | **+** | **+** | **+** | **-** | **-** | **-** | **-** | **+** |
| **Nitrilotriacetate monooxygenase component B (EC 1.14.13.-)** | **+** | **+** | **+** | **+** | **+** | **+** | **+** | **+** | **+** | **-** |
| **4-hydroxyphenylacetate 3-monooxygenase (EC 1.14.13.3)** | **-** | **-** | **+** | **+** | **+** | **-** | **-** | **+** | **-** | **-** |
| **P-hydroxybenzoate hydroxylase (EC 1.14.13.2)** | **-** | **-** | **-** | **-** | **-** | **-** | **+** | **-** | **-** | **-** |
| **Putative 4,5-dihydroxyphthalate dehydrogenase (pht3)** | **-** | **-** | **-** | **-** | **-** | **-** | **+** | **+** | **-** | **-** |
| **Isoquinoline 1-oxidoreductase (EC 1.3.99.16)** | **+** | **+** | **+** | **+** | **-** | **+** | **+** | **-** | **-** | **-** |
| **4-carboxy-2-hydroxymuconate-6-semialdehyde dehydrogenase(dmpD)** | **-** | **-** | **-** | **+** | **-** | **-** | **-** | **-** | **-** | **-** |
| **Benzoate-CoA ligase (EC 6.2.1.25)** | **-** | **-** | **+** | **+** | **-** | **-** | **-** | **-** | **+** | **-** |
| **Benzoate transport, inner-membrane translocator** | **+** | **+** | **+** | **+** | **+** | **-** | **-** | **-** | **-** | **-** |
| **Benzoyl-CoA-dihydrodiol lyase (EC 4.1.2.44)** | **+** | **-** | **+** | **+** | **+** | **-** | **-** | **-** | **+** | **-** |
| **Benzoyl-CoA oxygenase component B** | **+** | **-** | **+** | **+** | **-** | **-** | **-** | **-** | **+** | **-** |
| **Benzoate degradation ring-cleavage hydrolase** | **+** | **-** | **+** | **+** | **+** | **+** | **-** | **+** | **-** | **-** |
| **Benzoate 1,2-dioxygenase (EC 1.14.12.10)** | **-** | **-** | **+** | **+** | **-** | **-** | **-** | **-** | **-** | **-** |
| **3-oxoadipyl-CoA/3-oxo-5,6-dehydrosuberyl-CoA thiolase (PaaJ)** | **-** | **-** | **+** | **+** | **-** | **-** | **-** | **-** | **-** | **-** |
| **3-hydroxyadipyl-CoA dehydrogenase (PaaH)** | **+** | **-** | **+** | **+** | **-** | **+** | **-** | **-** | **-** | **+** |
| **Putative protein PaaI** | **+** | **-** | **+** | **+** | **+** | **-** | **-** | **-** | **-** | **-** |
| **4-hydroxybenzoyl-CoA reductase** | **-** | **-** | **+** | **+** | **+** | **+** | **-** | **+** | **-** | **-** |

Notes: 1. “+” and “-” indicate present and absent, respectively.

2. Numbers in brackets represent copy number of the gene in genomes.

Table S3. Cytochrome encoding genes involved in electron transport identified in the SAR324 cluster bacteria described in the present study (“-” indicates gene absence. Numbers represent the copy numbers of genes).

|  | **CTD7A-SAR324-1** | **CTD7A-SAR324-2** | **CTD7B-SAR324** | **CTD10-SAR324** | **GB-SAR324** | **SCGC AAA001-C10** | **JCVI-SC AAA005** | **SCGC AAA240-J09** | **SCGC AB-629-J17** | **SCGC AB-629-O05** |
| --- | --- | --- | --- | --- | --- | --- | --- | --- | --- | --- |
| **Cytochrome c' precursor/*C_553_*** | **1** | **-** | **2** | **1** | **-** | **1** | **-** | **-** | **-** | **1** |
| **Cytochrome c-554 precursor** | **1** | **-** | **2** | **1** | **1** | **-** | **1** | **1** | **-** | **1** |
| **Cytochrome c** | **1** | **1** | **1** | **1** | **1** | **-** | **3** | **1** | **1** | **-** |
| **Cytochrome c4 precursor** | **2** | **2** | **2** | **2** | **5** | **2** | **-** | **-** | **-** | **2** |
| **Cytochrome c6** | **-** | **-** | **1** | **1** | **-** | **-** | **-** | **1** | **-** | **-** |
| **Class III cytochrome C family protein** | **-** | **-** | **1** | **1** | **1** | **-** | **-** | **1** | **1** | **-** |
| **Cytochrome c551 peroxidase precursor** | **-** | **-** | **-** | **-** | **-** | **-** | **-** | **1** | **-** | **-** |
| **Cytochrome C assembly protein** | **1** | **1** | **1** | **1** | **1** | **1** | **1** | **1** | **1** | **-** |
| **Alternative cytochrome c oxidase subunit 2** | **1** | **1** | **1** | **1** | **1** | **-** | **1** | **1** | **-** | **-** |
| **Alternative cytochrome c oxidase subunit 1** | **1** | **1** | **1** | **1** | **2** | **-** | **1** | **-** | **-** | **-** |
| **Cytochrome c oxidase subunit 3** | **2** | **2** | **2** | **2** | **2** | **1** | **2** | **1** | **-** | **-** |
| **Cytochrome C oxidase, mono-heme subunit/FixO** | **-** | **-** | **-** | **1** | **-** | **-** | **-** | **-** | **-** | **-** |
| **Cytochrome oxidase maturation protein cbb3-type** | **-** | **-** | **-** | **1** | **-** | **-** | **-** | **-** | **-** | **-** |
| **Cytochrome c oxidase subunit 1 homolog, bacteroid** | **-** | **-** | **-** | **1** | **-** | **-** | **-** | **-** | **-** | **-** |
| **Cbb3-type cytochrome c oxidase subunit FixP** | **-** | **-** | **-** | **-** | **1** | **-** | **-** | **-** | **-** | **-** |
| **Gluconate 2-dehydrogenase cytochrome c subunit precursor** | **-** | **-** | **-** | **-** | **-** | **1** | **-** | **-** | **-** | **-** |
| **Alcohol dehydrogenase cytochrome c subunit precursor** | **-** | **-** |  | **-** | **-** | **-** | **1** | **-** | **-** | **-** |
| **Succinate dehydrogenase cytochrome b558 subunit** | **1** | **-** | **1** | **1** | **1** | **1** | **1** | **-** | **-** | **1** |
| **Cytochrome b(N-terminal)/b6/petB** | **-** | **-** | **-** | **-** | **-** | **-** | **1** | **-** | **-** | **-** |
| **L-lactate dehydrogenase [cytochrome]** | **-** | **-** | **-** | **-** | **-** |  | **2** | **-** | **-** | **-** |
| **Fructose dehydrogenase cytochrome subunit precursor** | **1** | **-** | **1** | **1** | **-** | **1** | **-** | **1** | **1** | **1** |
| **Cytochrome D1 heme domain protein** | **-** | **-** | **1** | **1** | **-** | **1** | **-** | **1** | **-** | **-** |
| **Polysulfide reductase (NfrD)** | **1** | **1** | **1** | **1** | **4** | **1** | **1** | **1** | **1** | **1** |
| **Sulfide dehydrogenase [flavocytochrome c](fcc)** | **1** | **1** | **-** | **1** | **-** | **-** | **-** | **-** | **-** | **-** |
| **Sulfate adenylyltransferase (EC 2.7.7.4)(sat)** | **-** | **-** | **1** | **-** | **2** | **-** | **1** | **1** | **-** | **-** |
| **Adenylylsulfate reductase subunit alpha** | **-** | **-** | **1** | **-** | **2** | **-** | **-** | **-** | **-** | **-** |
| **Adenylylsulfate reductase subunit beta** | **-** | **-** | **1** | **-** | **-** | **-** | **-** | **-** | **-** | **-** |
| **Adenosine-5'-phosphosulfate reductase beta subunit(apr)** | **-** | **-** | **-** | **-** | **1** | **-** | **-** | **-** | **-** | **-** |
| **Sulfite reductase, dissimilatory-type subunit alpha(dsrA)** | **-** | **-** | **-** | **-** | **1** | **-** | **-** | **-** | **-** | **-** |
| **Sulfite reductase, dissimilatory-type subunit beta(dsrB)** | **-** | **-** | **-** | **-** | **2** | **-** | **-** | **-** | **-** | **-** |
| **Sulfite reductase [ferredoxin]** | **-** | **-** | **1** | **1** | **-** | **-** | **-** | **-** | **-** | **-** |
| **heterodisulfide reductase, subunit E, putative** | **-** | **-** | **1** | **-** | **-** | **-** | **-** | **-** | **-** | **-** |
| **heterodisulfide reductase, subunit A** | **-** | **-** | **1** | **-** | **-** | **-** | **-** | **-** | **-** | **-** |
| **CoB--CoM heterodisulfide reductase subunit A** | **-** | **-** | **1** | **-** | **-** | **-** | **-** | **-** | **-** | **-** |

Table S4. Genes involved in chemotaxis response in the SAR324 cluster bacteria described in the present study.

|  | **CTD7A-SAR324-1** | **CTD7A-SAR324-2** | **CTD7B-SAR324** | **CTD10-SAR324** | **GB-SAR324** | **SCGC AAA001-C10** | **JCVI-SC AAA005** | **SCGC AAA240-J09** | **SCGC AB-629-J17** | **SCGC AB-629-O05** |
| --- | --- | --- | --- | --- | --- | --- | --- | --- | --- | --- |
| **Chemotaxis protein CheY** | **1** | **1** | **2** | **3** | **2** | **2** | **3** | **-** | **1** | **1** |
| **Chemotaxis response regulator protein-glutamate methylesterase of group 3 operon (CheB3)** | **-** | **-** | **-** | **-** | **-** | **-** | **2** | **-** | **-** | **1** |
| **Chemotaxis protein PomA** | **3** | **2** | **3** | **3** | **3** | **1** | **1** | **1** | **-** | **-** |
| **Chemotaxis response regulator protein-glutamate methylesterase of group 2 operon (CheB2)** | **-** | **-** | **-** | **-** | **-** | **-** | **1** | **1** | **-** | **-** |
| **Methyl-accepting chemotaxis protein II** | **-** | **-** | **-** | **-** | **2** | **-** | **1** | **-** | **-** | **-** |
| **Methyl-accepting chemotaxis protein III** | **-** |  | **-** |  | **-** | **-** | **1** | **-** | **-** | **-** |
| **Methyl-accepting chemotaxis protein IV** | **-** | **-** | **-** | **-** | **-** | **-** | **3** | **-** | **-** | **-** |
| **Chemotaxis protein CheA** | **-** | **-** | **-** | **-** | **2** | **-** | **4** | **-** | **-** | **-** |
| **Chemotaxis protein CheW** | **-** | **-** | **-** | **-** | **1** | **-** | **3** | **-** | **-** | **-** |
| **Chemotaxis protein methyltransferase(CheR)** | **-** | **-** | **-** | **-** | **1** | **-** | **3** | **-** | **-** | **-** |
| **Chemotaxis response regulator protein-glutamate methylesterase (CheB1)** | **-** | **-** | **-** | **1** | **-** | **-** | **2** | **-** | **-** | **-** |
| **purine-binding chemotaxis protein** | **-** | **-** | **-** | **-** | **-** | **-** | **1** | **-** | **-** | **-** |
| **Chemotaxis protein CheV** | **-** | **-** | **-** | **-** | **-** | **-** | **1** | **-** | **-** | **-** |
| **CheR methyltransferase, SAM binding domain** | **-** | **-** | **-** | **-** | **-** | **-** | **1** | **-** | **-** | **-** |
| **Chemotaxis protein CheY homolog** | **-** | **-** | **-** | **-** | **-** | **-** | **1** | **-** | **-** | **-** |
| **Chemotaxis protein LafU** | **2** | **1** | **2** | **1** | **1** | **-** | **-** | **-** | **-** | **-** |
| **Methyl-accepting chemotaxis protein PctC** | **-** | **-** | **-** | **-** | **1** | **-** | **-** | **-** | **-** | **-** |

Note: “-” indicates absence of the gene and numbers show copy number of genes.


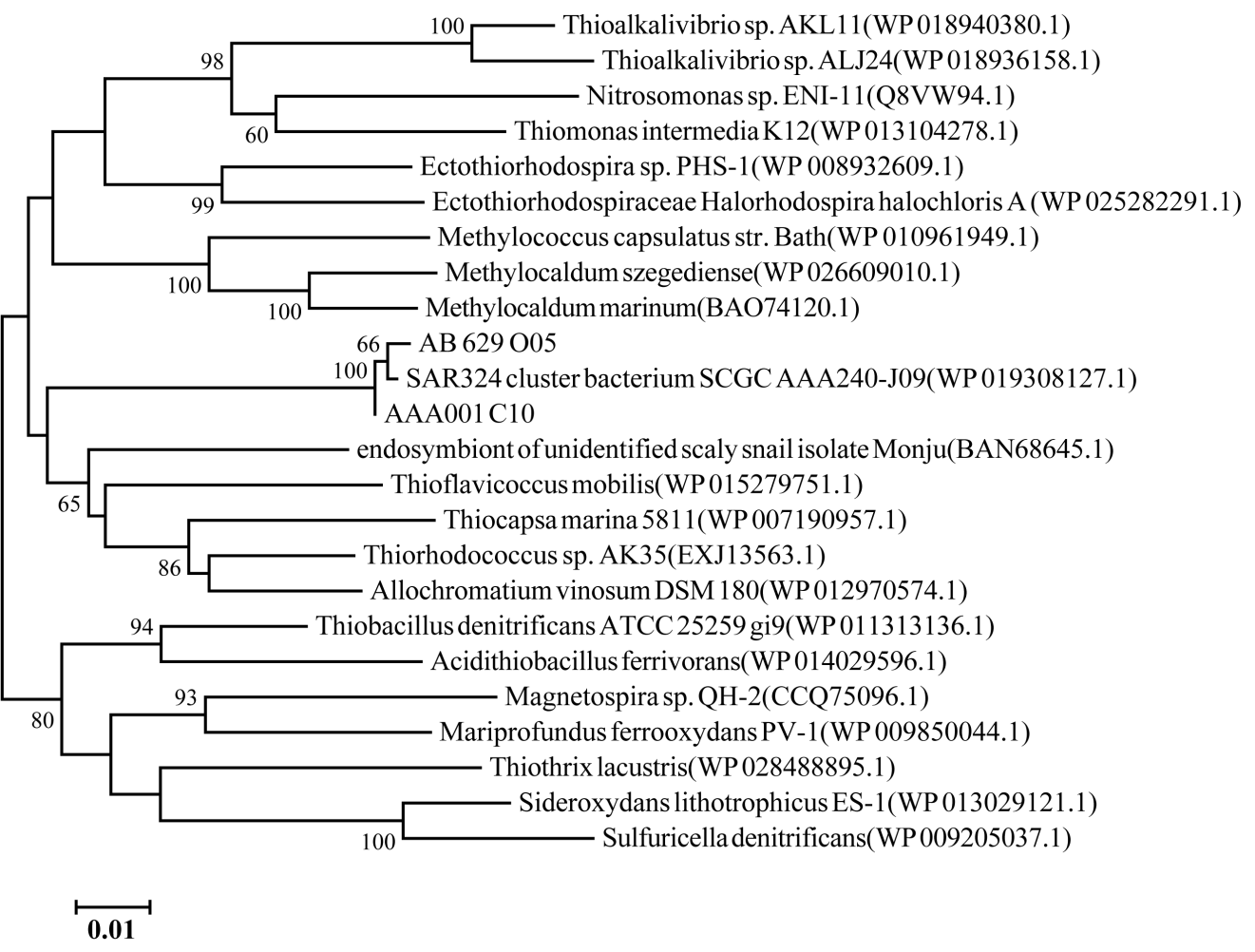


**A.**


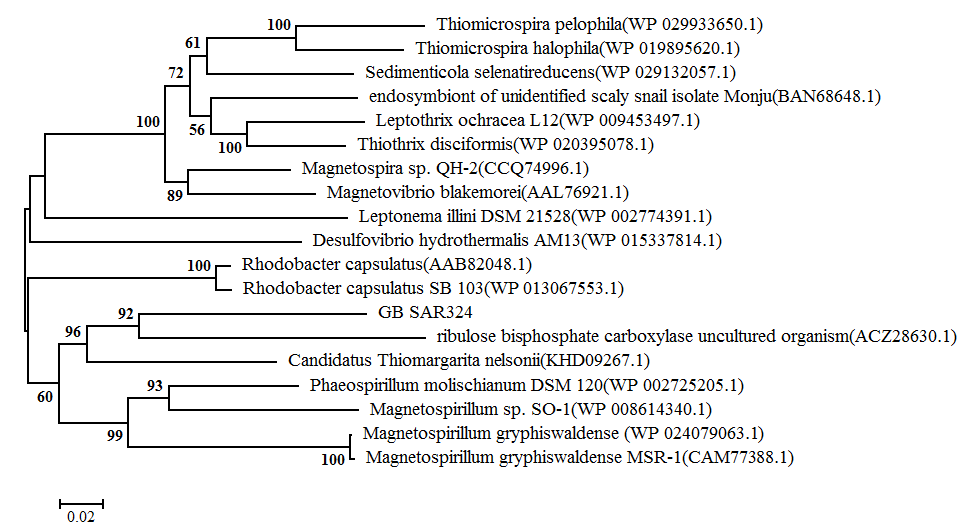


**B.**

Figure S1. Phylogenetic tree based on the DNA sequences of *cbbL* (A) and *cbbM* (B) retrieved from the genomic sequences of SAR324 cluster bacteria.


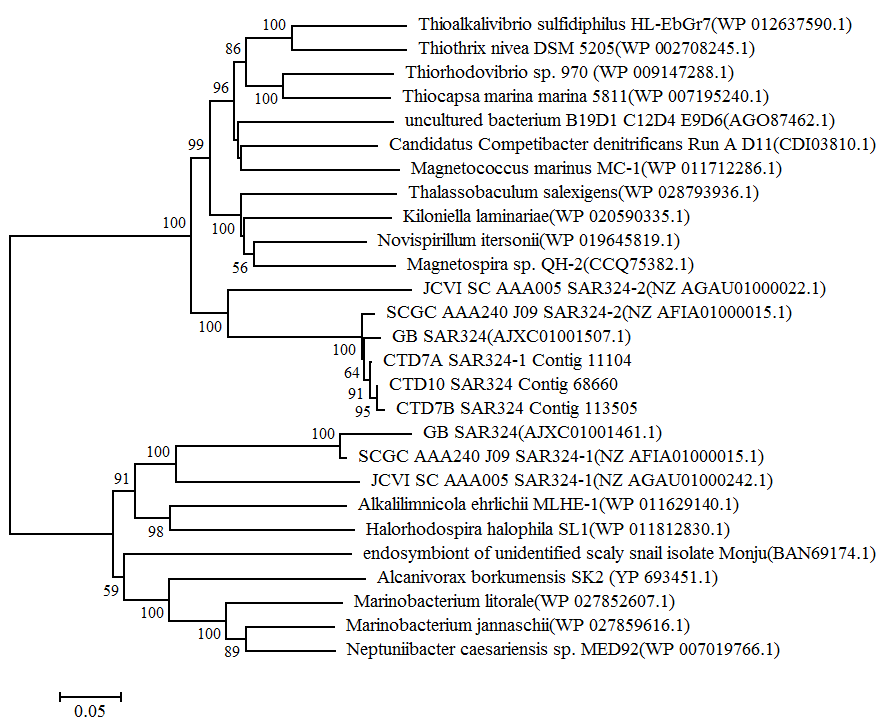


Figure S2. Phylogenetic organization based on the DNA sequence of acetyl-CoA synthase gene retrieved from the genomic sequences of SAR324 cluster bacteria.


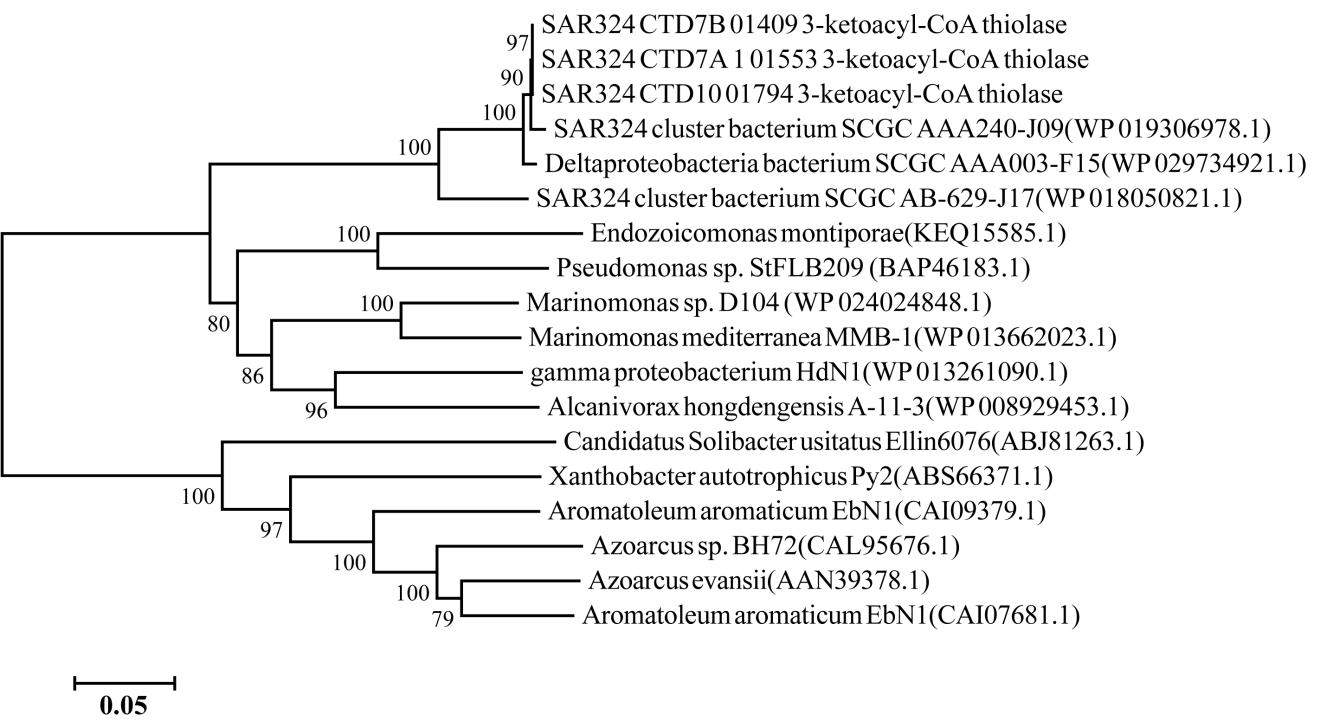


Figure S3. Phylogenetic tree based on the DNA sequences of 3-ketoacyl-CoA thiolase gene retrieved from the genomic sequences of SAR324 cluster bacteria.


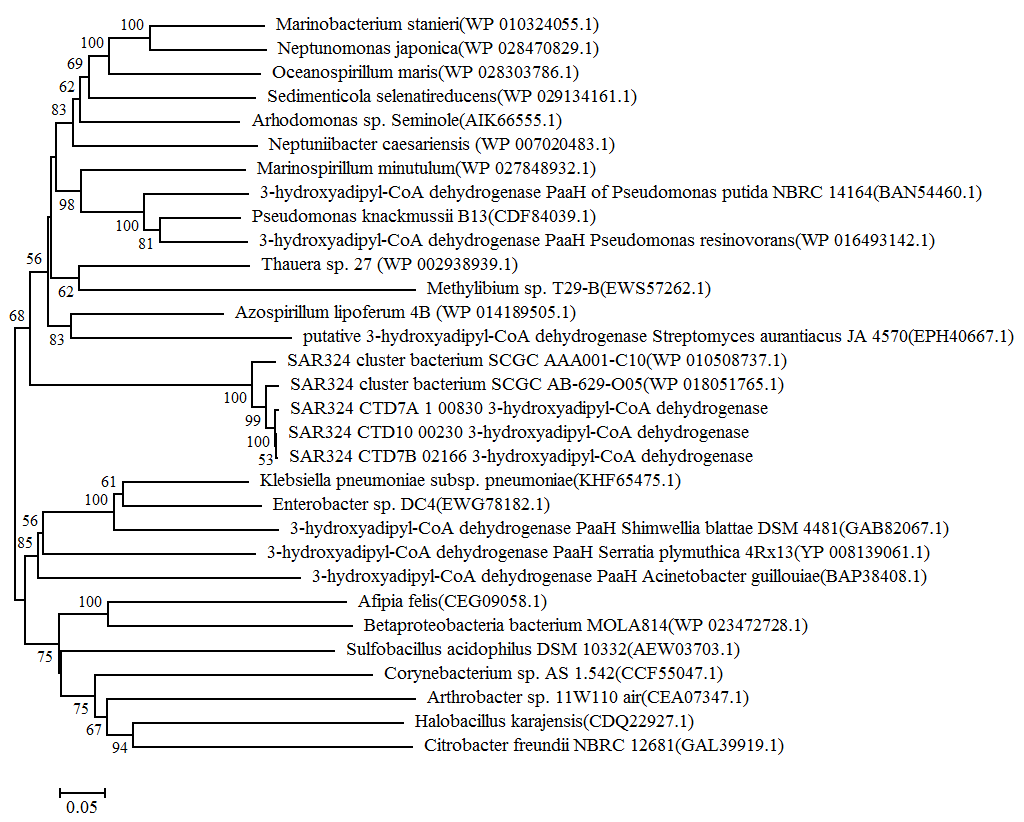


Figure S4. Phylogenetic tree based on the DNA sequences of 3-hydroxyadipyl-CoA dehydrogenase (PaaH) gene retrieved from the genomic sequences of SAR324 cluster bacteria. The phylogenetic organization was constructed using the maximum likelihood (ML) method in the MEGA program and bootstrap values at nodes were obtained from 100 replicates.


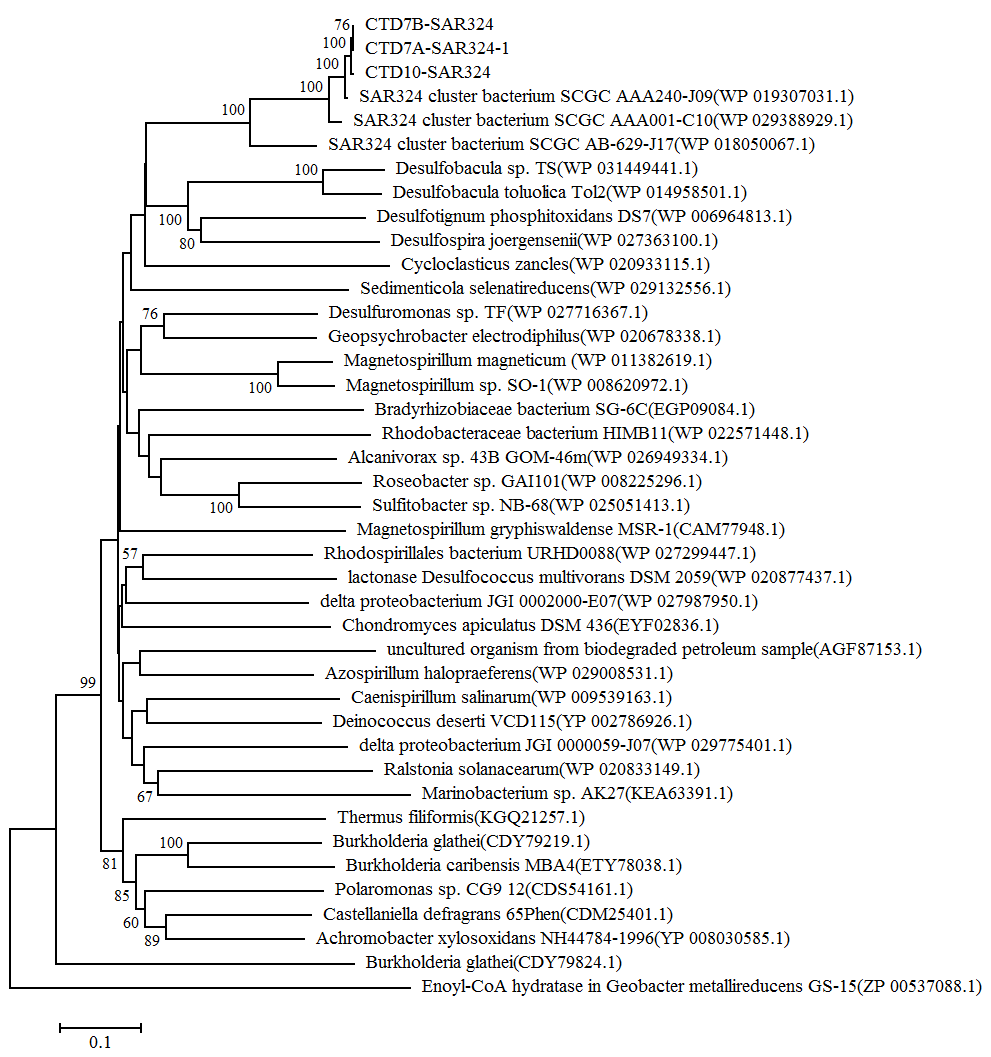


Figure S5. Phylogenetic tree based on the DNA sequences of benzoate degradation ring-cleavage hydrolase gene retrieved from the genomic sequences of SAR324 cluster bacteria.


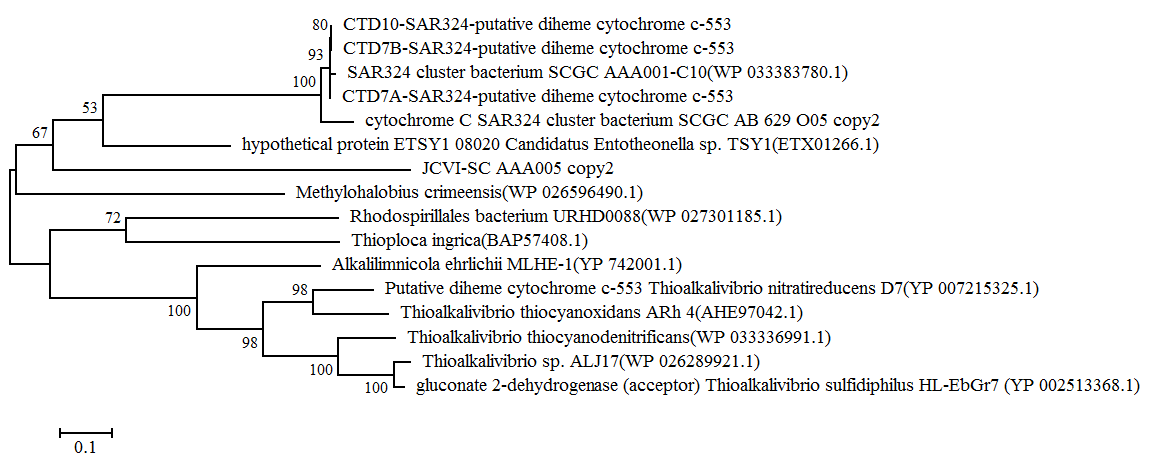


Figure S6. Phylogenetic tree based on the DNA sequences of cytochrome *C_553_* gene retrieved from the genomic sequences of SAR324 cluster bacteria.


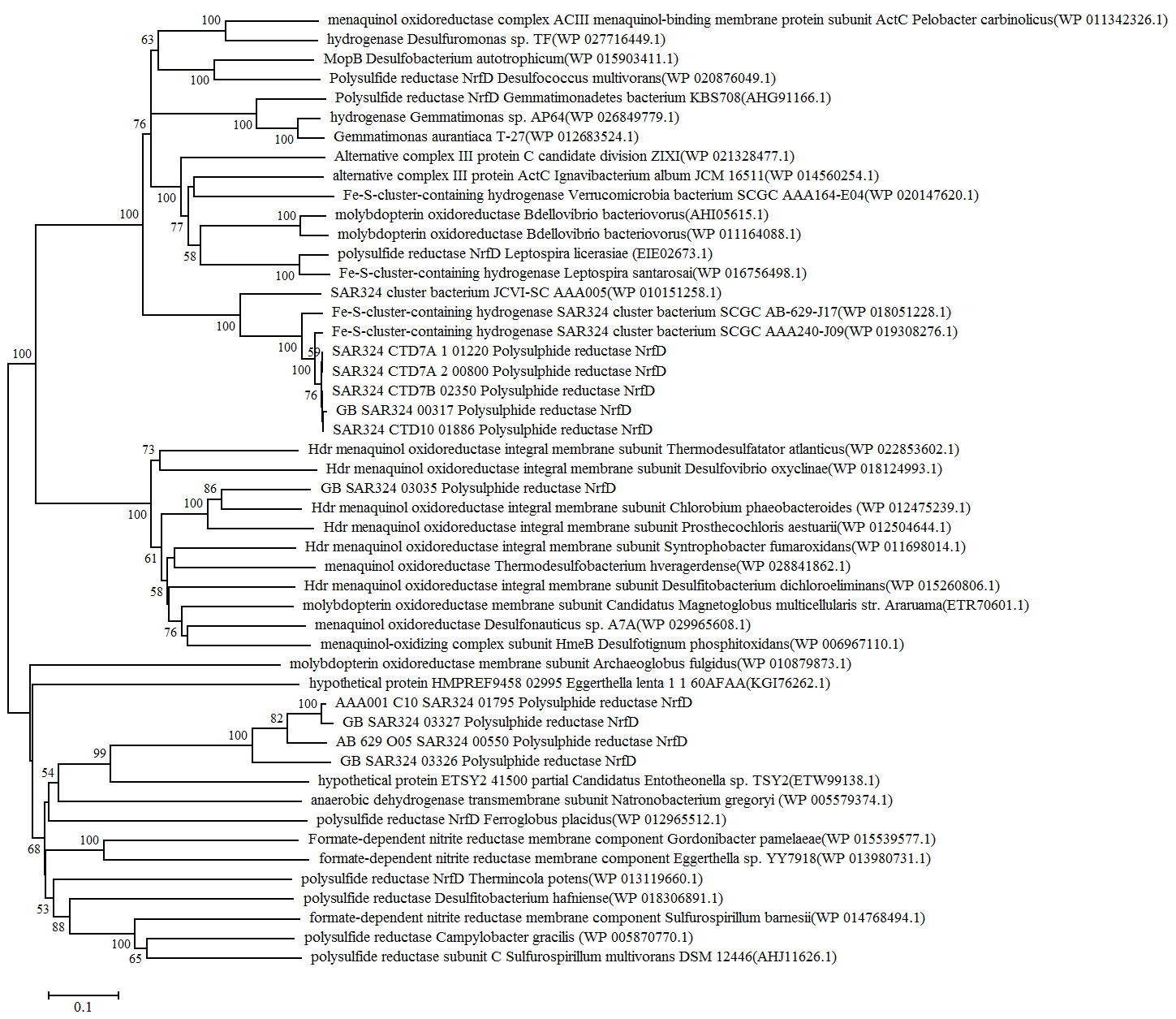


Figure S7. Phylogenetic tree based on the DNA sequences of polysulfide reductase (NfrD) gene retrieved from the genomic sequences of SAR324 cluster bacteria.


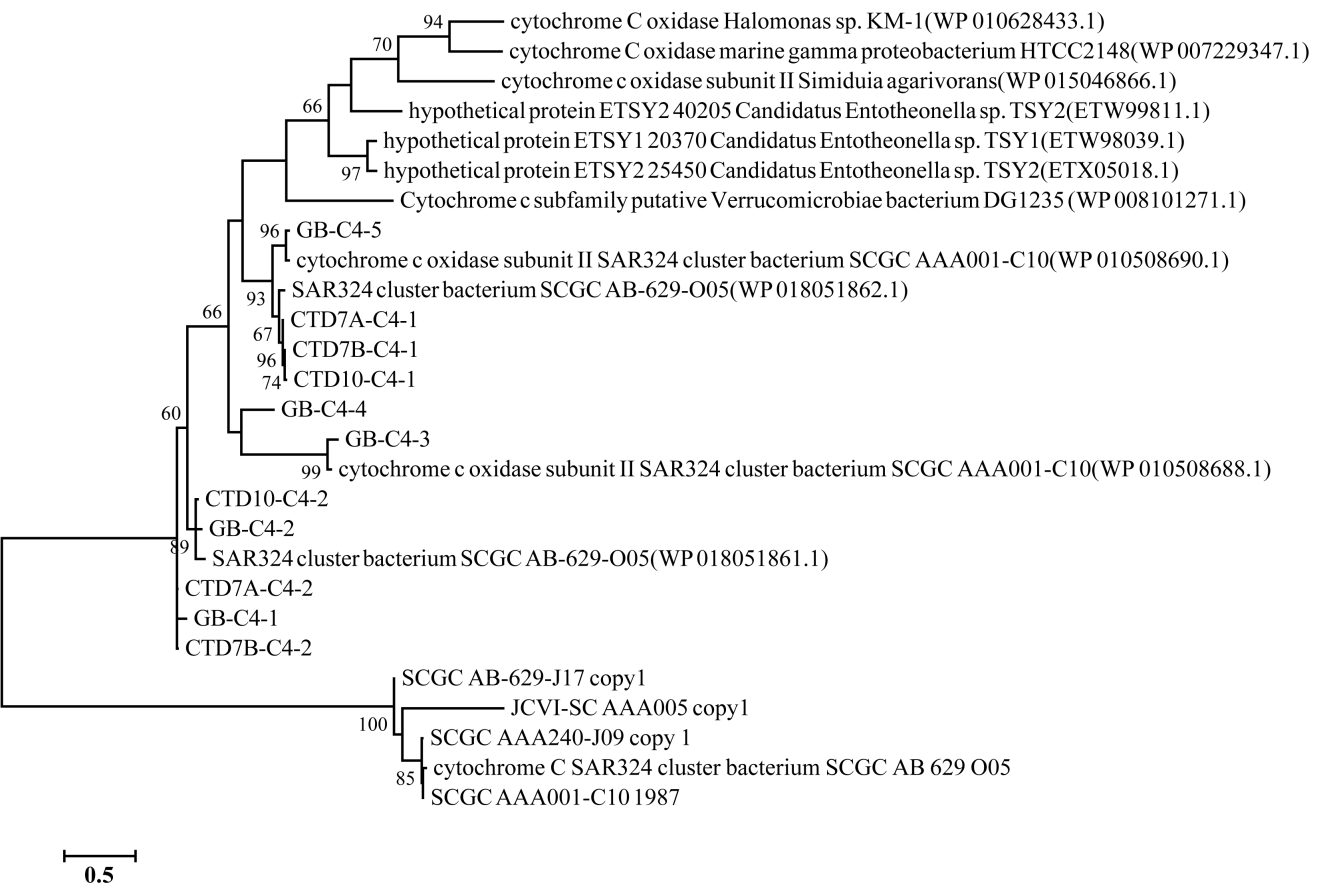


Figure S8. Phylogenetic tree based on the DNA sequences of cytochrome *C_4_* gene retrieved from the genomic sequences of SAR324 cluster bacteria.


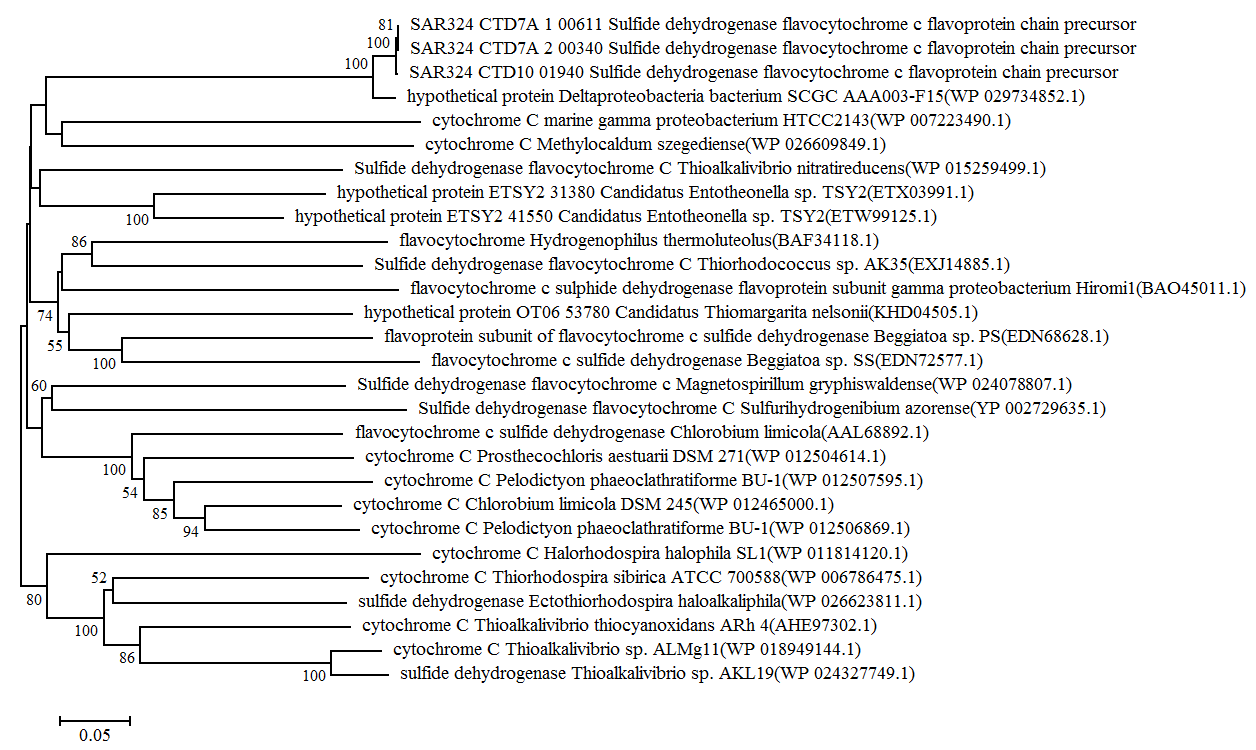


Figure S9. Phylogenetic tree based on the DNA sequences of sulfide dehydrogenase (flavocytochrome C) gene retrieved from the genomic sequences of SAR324 cluster bacteria.


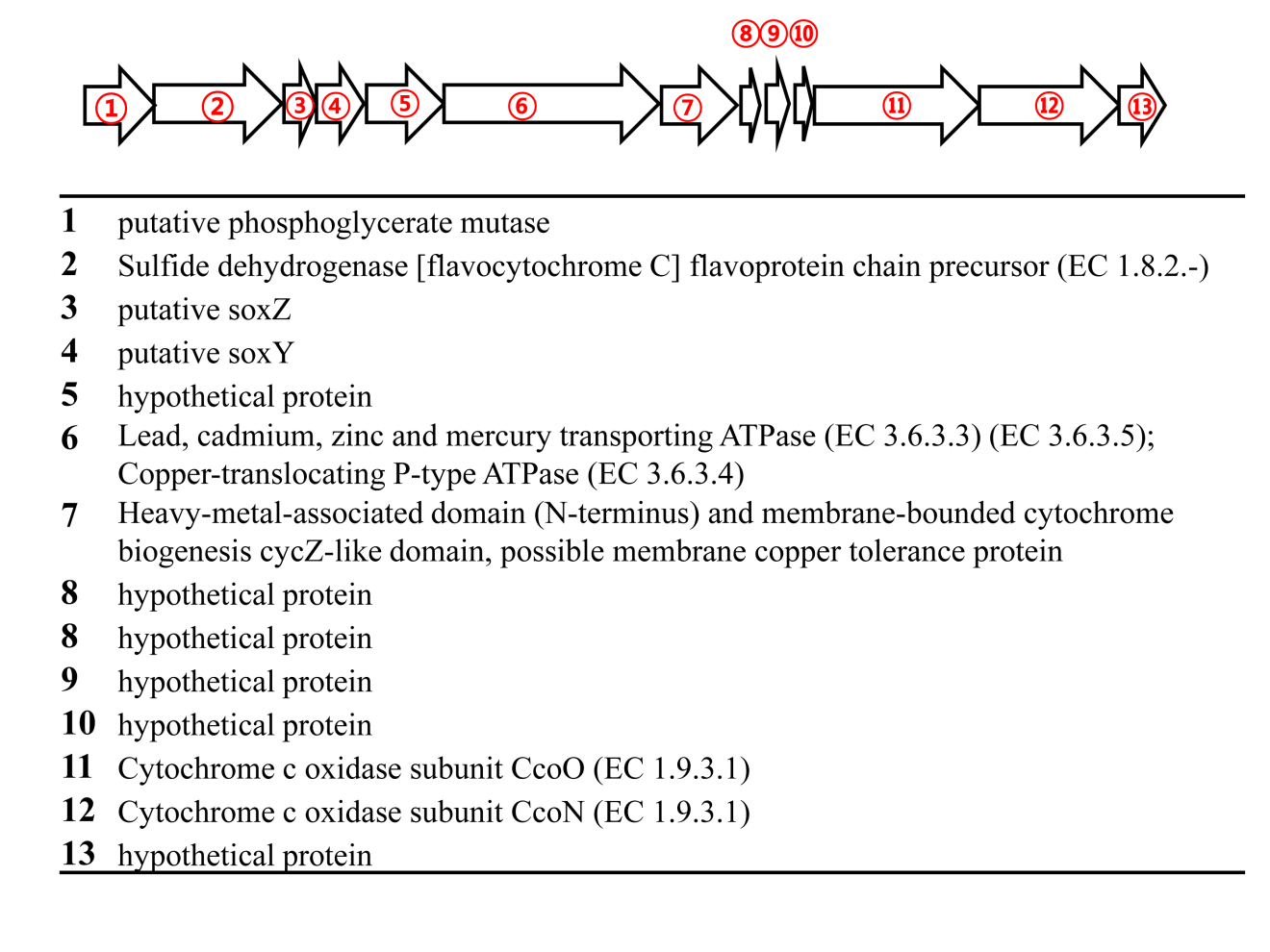


Figure S10. The sulfide dehydrogenase (flavocytochrome C) gene cluster in CTD10-SAR324 genomic bin.


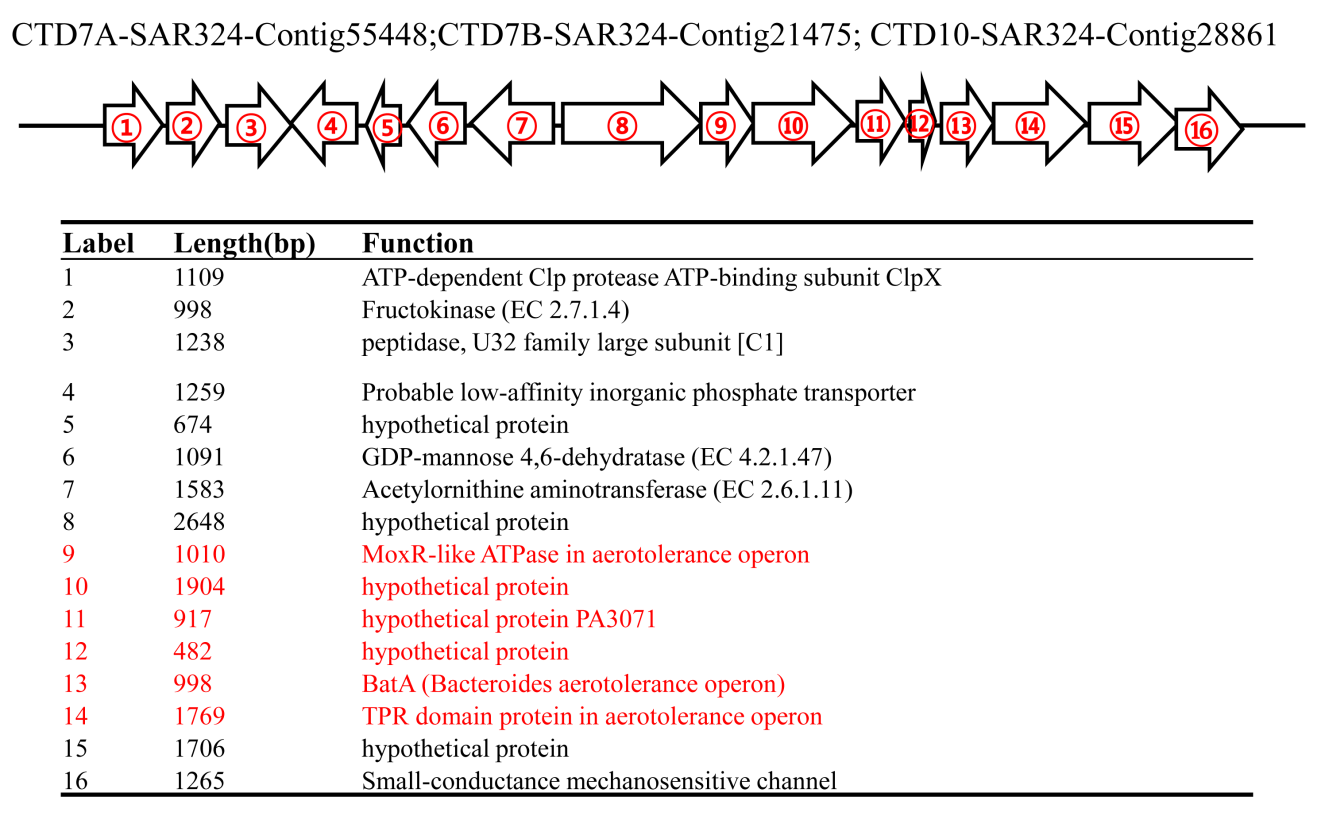


Figure S11. The aerotolerance operon in CTD10-SAR324 genomic bins from the present study.
